# Lateral unicompartmental knee arthroplasty anatomy, indications, technique, and outcomes: a narrative review

**DOI:** 10.1007/s00402-025-06157-4

**Published:** 2025-12-15

**Authors:** Jennifer Hong, Paul Tjoumakaris, Sahil Sanghavi, Ahab Alnemri, Praneeth Thota, Weston Smith, Emily Eiel, Neil Sheth

**Affiliations:** 1 University of Pennsylvania, Philadelphia, USA; 2 Sancheti Institute For Orthopaedics & Rehabilitation, Pune, India

**Keywords:** Unicompartmental knee arthroplasty, Arthroplasty, Knee osteoarthritis, Functional outcomes, Implant survivorship, Isolated compartment arthrosis

## Abstract

Lateral unicompartmental knee arthroplasty (UKA) is an effective surgical option for isolated lateral compartment osteoarthritis, though it remains less common than medial UKA. The lateral compartment differs substantially from the medial compartment in osseous morphology, meniscal mobility, and reliance on soft tissue stabilizers, resulting in unique kinematics that require distinct implant designs and surgical strategies. While earlier guidelines delineated narrow indications, contemporary evidence supports expanded indications, with good outcomes even in younger patients, those with higher body mass index, or mild patellofemoral joint disease. Technical considerations include surgical approach, alignment goals, and implant choice, with fixed-bearing implants preferred due to lower dislocation risk and robotic-assisted techniques showing promise for optimizing implant positioning. Modern series demonstrate survivorship exceeding 90% at 10–15 years, with functional outcomes comparable to medial UKA and superior to total knee arthroplasty in some areas such as recovery, patient satisfaction, and wound infection and other complication rates. This review summarizes the anatomy and biomechanics of the lateral compartment of the knee, indications, surgical technique, implant options, and clinical outcomes of lateral UKA.

## Introduction

Partial or unicompartmental knee arthroplasty (UKA) is increasing in prevalence, especially as more primary arthroplasty procedures have moved to the outpatient setting. UKAs constitute approximately 10% of all primary knee arthroplasty [1]. Of all UKA procedures, lateral UKA for the treatment of isolated lateral compartment disease comprises about 10% [2]. The tenfold lower adoption of lateral UKA compared to medial UKA is partially due to the relatively lower prevalence of isolated lateral compartment arthritis, compounded by limited surgeon familiarity and historical concerns about increased complexity and risk [3]. However, recent studies have demonstrated that compared to total knee arthroplasty (TKA), lateral UKA produces better short-term patient-reported outcome measures (PROMs) and fewer complications, is more easily performed in the outpatient setting, and better restores native knee kinematics [4–6].

Lateral UKA is considered by many surgeons to be riskier and more technically challenging than medial UKA [2]. The lateral compartment of the knee has different anatomic and kinematic properties from the medial compartment which require different reconstructive considerations (e.g. alignment, component rotation, and soft-tissue balance) that are critical to long-term implant longevity and functional outcomes [7]. Therefore, lateral UKA should be considered a separate entity from medial UKA. This review provides a brief overview of the anatomy and biomechanics of the lateral compartment of the knee, emerging indications, surgical technique, and mid- to long-term clinical outcomes of lateral UKA.

### Anatomy and biomechanics of the lateral compartment

The medial and lateral compartments of the knee differ greatly in bony and soft-tissue anatomy and biomechanics. The lateral tibial plateau is 2–3 mm more proximal than the medial tibial plateau, and lateral tibial cartilage is thicker than medial cartilage by approximately 2 mm, which may contribute to the relative rarity of isolated lateral compartment osteoarthritis (OA) [8]. The medial tibial plateau is concave and allows the convex medial femoral condyle to rest inside it, forming a stable, constrained structure. On the other hand, the lateral tibial plateau is convex and also articulates with a convex lateral femoral condyle, giving the lateral compartment less stability [7]. The lateral meniscus helps stabilize the lateral compartment by providing a concave surface for articulation with the lateral femoral condyle. However, the lateral meniscus must accommodate the relatively greater range of motion in the lateral compartment, and it does so through its C shape and less restraining static attachments to the tibia compared to the medial meniscus. This inherent mobility of the lateral compartment means that it depends more on soft-tissue stabilization, and the lateral ligamentous structures of the knee experience greater forces than the medial structures during normal gait [7]. The most important stabilizing structures include the lateral collateral ligament (LCL), popliteus tendon, popliteofibular ligament, and posterior cruciate ligament (PCL) [9]. These structures prevent varus gapping, external and internal rotation, and posterolateral tibial translation.

These anatomic differences produce different kinematic characteristics of the two compartments. In normal knee alignment, the mechanical axis of the lower extremity lies slightly medial to the center of the knee, causing the medial compartment to experience greater compressive force (60% of the load) while the lateral compartment experiences greater tension [7]. During knee flexion, the lateral femoral condyle translates posteriorly approximately 21 mm while the medial condyle acts as a pivot point with only 1.9 mm of translation, causing net external rotation of the femur [10]. When the knee extends, the reverse occurs and the femur internally rotates on the tibia, known as the “screw-home” mechanism of the knee [11].

These biomechanical differences distinctly affect the design of lateral UKA implants. The soft tissues and dimensions (e.g. anteroposterior-to-mediolateral ratio) of the lateral compartment contribute to the implant sizing, position, and surgical technique. Lateral UKA components typically incorporate flatter articular surfaces with lower conformity and greater rotational allowance. They must accommodate for larger posterior femoral rollback to avoid over-constraining the lateral compartment and disrupting the screw-home mechanism [12]. Furthermore, due to the more delicate kinematics of the lateral component, lateral UKA is particularly sensitive to surgical technique and requires great precision to ensure proper component positioning and soft-tissue balancing. This will be discussed in greater detail in the Surgical Techniques section.

#### Development of lateral compartment osteoarthritis

Malalignment of the femoral side of the knee joint contributes significantly to the development of lateral compartment OA. In normally aligned knees, the femoral condyles are at a mean of 83.3° to the anatomical femoral axis (6.7° of valgus) [13]. This angle is unchanged in knees with varus misalignment and medial compartment OA, but is reduced to a mean of 80.7° in valgus knees with lateral compartment OA [13]. This femur deformity overloads the lateral compartment relative to the medial compartment, leading to primary lateral compartment OA. Each increase in valgus malalignment of 1° increases one’s risk of lateral compartment OA by 55%, with greater malalignment associated with greater functional decline [14].

On the tibial side of the joint, external tibial torsion is associated with lateral OA [15]. Overall, the tibial side of the joint contributes less to the development of primary lateral OA than the femoral side. However, it is more often the cause of post-traumatic secondary OA, particularly in the setting of meniscectomy or tibial plateau fracture. Damage to the lateral meniscus, which normally stabilizes the joint and attenuates shock, also increases risk of OA by reducing contact area and increasing stress on the articular cartilage. Lateral meniscectomy is associated with greater increase in risk of OA than medial meniscectomy [16]. In contrast to medial compartment OA, in which most wear is located centrally and anteriorly, lateral compartment OA demonstrates a more posterior wear pattern [15].

#### Inter-individual differences

Variation in knee geometry and kinematics between individuals may contribute to different patient outcomes. Medial posterior tibial slope (MPTS), lateral posterior tibial slope (LPTS), and medial proximal tibial angle (MPTA) vary considerably between and within individuals [17]. A study based on CT scans of European patients undergoing TKA revealed that MPTS and LPTS vary between individuals with ranges over 20°, while MPTA varies with a range over 12° [18]. The intra-individual difference between MPTS and LPTS averages 2.6° but may reach a maximum of 9.5° [18]. Due to these differences, knee arthroplasty may lead to alterations in knee anatomy and alignment, a consideration that is particularly relevant in cruciate ligament-preserving surgeries such as UKA, in which excessive alteration of posterior tibial slope may predispose to degeneration and rupture of the anterior cruciate ligament [19].

### Indications and patient evaluation

Several years after lateral UKA was first introduced by Skolnick and colleagues in 1975 with a small case series [20], Kozinn and Scott published a set of indications for UKA [21]. These traditional indications included unicompartmental OA or focal osteonecrosis of the lateral compartment of the knee in a patient with age above 60 years, weight below 82 kg, at least 90° of flexion, less than 5° flexion contracture, less than 15° angular deformity, intact anterior cruciate ligament (ACL), and absence of heavy physical labor [21]. Traditional contraindications included disease in the other compartment or patellofemoral joint (PFJ), chondrocalcinosis, and inflammatory arthritis. Furthermore, valgus deformity of the knee should be passively correctable to neutral alignment, nor should it be over-correctable into varus, which may suggest excessive lateral soft-tissue laxity or the presence of medial compartment disease.

Over the years, however, improved UKA implant designs and surgical techniques have expanded indications for lateral UKA. In one prospective series of one thousand UKAs, Pandit and colleagues found that fewer than one-third of UKAs were performed in patients considered to be “ideal” according to the classic Kozinn and Scott criteria, and survivorship did not differ between UKAs with and without contraindications [22]. Studies have found variable effects of age, obesity, flexion contracture, PFJ disease, chondrocalcinosis, and ACL deficiency on lateral UKA outcomes and survivorship. Studies examining the effects of age, obesity, and PFJ disease are summarized in Table 1. More long-term studies are needed to elucidate the significance of these factors in pre-operative evaluation for lateral UKA. According to currently published data, age under 60, elevated body mass index (BMI), and mild PFJ disease are no longer considered strict contraindications to lateral UKA in contemporary practice.

#### Age

Younger patients tend to have greater demand for activity, which may result in greater implant wear and loosening and higher revision rate. Data from the Dutch Arthroplasty Register revealed that lateral UKAs performed on patients younger than 60 years had higher 5-year revision rate than those on patients older than 60 (15.4% versus 9.75%) [23]. A cohort study of 40 younger patients (mean age 57.6 years, range 40–68), however, found excellent outcomes as measured by the Oxford Knee Score in 80% of patients and 93.1% survivorship at 10-year follow-up [24]. Similar results were found by several other studies (Table 1). These findings suggest that although younger patients may face earlier revision, favorable outcomes and the ability of lateral UKA to restore high levels of activity make it a worthwhile treatment in this population.

#### Obesity

In a cohort of 100 lateral UKAs, Giordano and colleagues found excellent PROMs in both patients with BMI above 30 kg/m^3^ and those with BMI below 30, suggesting that obesity should not be considered a contraindication for lateral UKA [25]. Similar results were found by several other cohort studies (Table 1). Van der List and colleagues showed that although functional outcomes are poorer in obese patients for both TKA and lateral UKA, the correlation is stronger for TKA than lateral UKA [5].

#### PFJ disease

Data examining the impact of PFJ disease on lateral UKA outcomes are relatively sparse. Kennedy and colleagues found in a cohort of 325 knees, including 46 with full-thickness cartilage loss in the PFJ, that post-operative PROMs, Tegner activity score, revision rate, and survivorship did not differ vary with the pre-operative state of the PFJ [26]. Similarly, mid-term data on 140 knees by Burger and colleagues demonstrated that mild to moderate pre-operative PFJ OA and malalignment does not significantly impact post-operative PROMs [27]. Plancher and colleagues also found that post-operative in Knee Injury and Osteoarthritis Outcome Score (KOOS) and Kujala scores did not vary significantly between patients with and without severe lateral facet PFJ OA undergoing lateral UKA [28]. There was also no significant difference in survivorship (18.9 years in patients with severe lateral facet PFJ OA versus 16.6 years in those without) [28]. These excellent outcomes in patients with PFJ OA may be due to realignment of the patella and redistribution of mechanical load across the PFJ during lateral UKA.

#### ACL insufficiency

The effect of ACL insufficiency on outcomes of lateral UKA has not been as well-studied as its effect on outcomes of medial UKA. A cohort study including 57 lateral UKAs by Plancher and colleagues found no significant difference in KOOS or 10-year survivorship between ACL-deficient and ACL-intact knees [29]. However, a more recent matched-pair analysis later published by the same group revealed that 10-year survivorship in the ACL-deficient group was 85%, significantly lower than that of the ACL-intact group, which was 100% [30]. However, there was still no statistically significant difference in patient-reported outcomes between the groups [30]. More research is needed to corroborate these findings.

#### Radiologic and intra-operative evaluation

In addition to standard pre-operative knee radiographs, full-length radiographs are recommended to assess overall alignment of the lower extremity. Alongside standard anteroposterior (AP) and lateral radiographs, the posteroanterior (PA) flexion (Rosenberg) view is valuable as AP radiographs underestimate bone loss in valgus knees [2]. Valgus stress radiographs can be used to evaluate the correctability of varus and predict postoperative coronal alignment [31]. Magnetic Resonance Imaging (MRI) can help provide information regarding the medial meniscus and cartilage defects, particularly if signs or symptoms from other compartments are present. The final decision to perform a lateral UKA or TKA is made intra-operatively, after examining the medial and patellofemoral compartments and surrounding structures. Surgeons must be ready to convert to a TKA intra-operatively when indicated.

### Surgical technique

Lateral UKA is considered by many surgeons to be more technically challenging than medial UKA. Anatomic and kinematic differences between the medial and lateral compartments introduce important variations in surgical technique, and the relative rarity of the procedure for most surgeons compounds its difficulty. Key technical distinctions from medial UKA include choosing a lateral or medial parapatellar approach, intentionally undercorrecting valgus deformity in lateral UKA (as opposed to achieving neutral or slight varus alignment in medial UKA), and accounting for a distinct wear pattern and risk of patellar impingement by precise component positioning. Specifics of these topics are detailed in the following sections.

#### Approach

Two main surgical approaches, lateral parapatellar and medial parapatellar, are used for lateral UKA. A comparative study by Edmiston and colleagues demonstrated no difference in survivorship after the two approaches, but the lateral approach resulted in better post-operative flexion [32]. The lateral parapatellar approach offers the advantages of a shorter incision, direct visualization, rapid recovery of quadriceps function, and preservation of the medial side of the knee and its vascular supply. However, with the lateral parapatellar approach, there is a susceptibility to position the tibial component in external rotation, which is associated with poorer functional outcomes and should be avoided [33]. Additionally, if a future revision is required, the most lateral of the existing incisions should be used because the blood supply of the periarticular soft tissues arises from the medial side of the knee. In the case of a prior lateral approach, this demands a revision also through the lateral approach, which is more challenging [12, 34].

On the other hand, most surgeons are more familiar with the medial parapatellar approach, which also allows easier conversion to a TKA if required intra-operatively. However, the medial approach risks damage to the quadriceps complex and medial meniscus and may not allow for adequate exposure of the lateral compartment [35].

#### Alignment

Patients undergoing lateral UKA have some degree of pre-operative valgus deformity. This valgus deformity should be slightly undercorrected in lateral UKA, although the exact amount of undercorrection to optimize outcomes is debated. Van der List and colleagues [36] and Zheng and colleagues [37] both demonstrated that patients with post-operative valgus alignment of 3° or more attained better functional outcomes than those with more neutral alignment. Ohdera and colleagues had previously also recommended aiming for 5° to 7° of post-operative valgus [38]. In contrast, Vossen and colleagues evaluated the pre-operative and post-operative Coronal Plane Alignment of the Knee (CPAK) distribution in 305 knees undergoing lateral UKA to assess the correlation of phenotype restoration or variation with PROMs [39]. They found no association between better post-operative PROMs and restoration of pre-operative phenotype.

#### Component positioning

Proper component congruency is more difficult to achieve in lateral UKA compared to medial UKA. The pattern of wear in lateral compartment disease is more posterior, leaving residual cartilage on the distal femoral condyle [2]. To prevent patellar impingement, Scott recommends careful removal of cartilage and undersizing of the femoral component anteriorly [2]. Lateral UKA also carries a greater risk of alignment overcorrection and greater difficulty in predicting post-operative alignment using computer navigation [40]. Due to the screw-home mechanism, femoral implant positioning in flexion should exaggerate external rotation and lateral positioning to avoid impingement of the tibial spine in extension and maximize mediolateral congruency [2, 37]. Conversely, the components must not be placed too laterally when in extension to avoid overloading the lateral part of the tibial plateau when the knee is flexed to 30° [37]. When performing the sagittal tibial cut, internal rotation of the tibial component must also accommodate the screw-home mechanism [37].

#### Robotic-assisted surgery

Robotic arm-assisted surgery has recently been applied to UKA to assist surgeons with implant positioning. Although early data on functional outcomes, survivorship, and patient satisfaction after robotic-assisted medial UKA are mixed, many recent studies report outcomes mildly improved or similar to conventional surgery [41–43]. Data on robotic arm-assisted lateral UKAs are relatively sparse, but early studies have shown promising results. Ruderman and colleagues demonstrated 96.1% survivorship, high satisfaction, and low pain scores among 77 robotic-assisted lateral UKAs at 10.2 years, marking the longest follow-up to date [44]. A two-center cohort study by Zambianchi and colleagues demonstrated 100% survivorship among 67 robotic-assisted lateral UKAs and significantly improved clinical scores at 3-year follow-up [45]. Another short-term study by Batailler and colleagues also demonstrated 100% survivorship among 23 knees at 2.1-year follow-up [46]. A comparative study of 52 robotic-assisted lateral UKAs and 43 conventional lateral UKAs by Maritan and colleagues demonstrated no significant differences in any clinical score or survivorship at 7.5-year follow-up [47]. Canetti and colleagues demonstrated significantly quicker return to sports with robotic-assisted technique compared to conventional technique [48]. The results of these studies are consistent with those of a meta-analysis which found that robotic-assisted surgery is associated with decreased risk of aseptic loosening, which is one of the most common reasons for implant failure [49]. Robotic-assisted surgery may be especially helpful in improving component alignment in lateral UKA given the low tolerance for malalignment in this compartment. Robotic-assisted lateral UKA has been found to produce fewer limb alignment outliers (26%) than conventional technique (61%), facilitate intra-operative assessment of soft tissue balance, and improve femorotibial component positioning to better match native knee alignment [50–52]. Further studies are needed to assess the long-term survivorship and modes of failure among robotic-assisted technique compared to conventional technique. Additionally, the widespread adoption of robotic-assisted surgery has been precluded by cost, learning curve, and technology availability, in addition to persistent concerns about increased operative time and cost-effectiveness.

### Implant options

Implant options for lateral UKA can be broadly categorized by bearing type and fixation method, each of which influences surgical technique and patient outcomes. Fixed-bearing implants are currently preferred over mobile-bearing implants due to superior survivorship and lower risk of bearing dislocation [53]. Cemented fixation has been the traditional approach, whereas cementless fixation has demonstrated encouraging early outcomes [54, 55].

#### Mobile-versus fixed-bearing implants

**Fig. 1:**
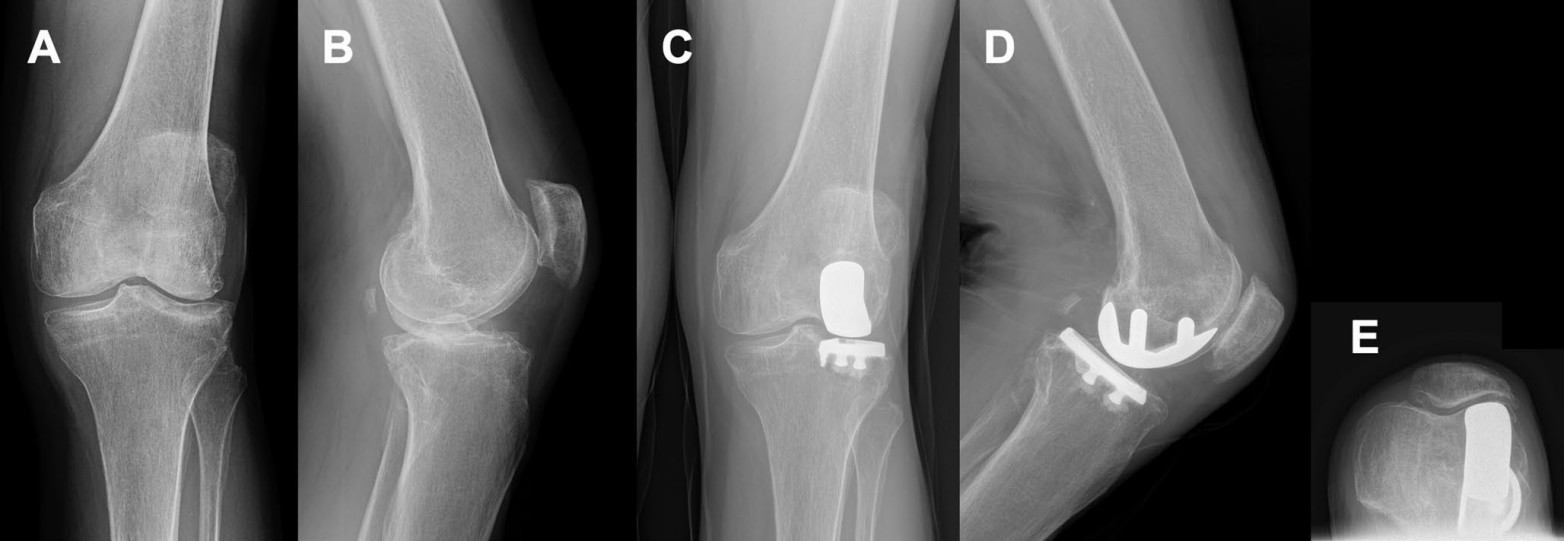
72-year-old female with long-standing left lateral knee pain. **A** Anteroposterior and **B** lateral images demonstrate isolated lateral compartment degenerative joint disease with a posterolateral wear pattern. After failing conservative treatment, she was a candidate for a left lateral UKA. **C** Anteroposterior, **D** lateral, and **E** merchant views at 4-year follow-up

The selection of bearing type represents a critical decision in lateral UKA. While fixed bearings rely on conformity and wear resistance, mobile bearings aim to reduce contact stress through self-alignment. Mobile-bearing femoral components have a spherical sagittal shape with a continuous radius of curvature, allowing fewer size options but maximum congruency. Fixed-bearing designs allow for more anatomic femoral components with multiple sizes that better replicate native anatomy (Fig. 1). These differences lead to distinct failure modes and survivorship rates.

Although mobile-bearing designs were theoretically developed to reduce polyethylene wear [56], studies have not confirmed this benefit and in fact suggested higher wear rates compared to fixed-bearing designs [57, 58]. Meta-analysis has shown that fixed-bearing metal-backed implants have lower failure rates (0.8%) compared to mobile-bearing metal-backed (7.1%) implants among studies with minimum 2-year follow-up [53]. Bearing dislocation is the most common cause of early failure (< 5 years) and the most common failure mode in mobile-bearing implants (27%), while OA progression is the most common failure mode in fixed-bearing implants (44%) [59]. Mobile bearings have a four-fold higher risk of revision than fixed bearings in lateral UKAs [60]. Due to the greater femoral rollback and different kinematics in the lateral compartment, mobile-bearing implants carry a higher risk of bearing dislocation compared to medial UKA [61]. A 2025 cohort of 115 lateral mobile-bearing UKAs by Walker and colleagues with long-term follow-up demonstrated 74.8% survivorship after 10 years, significantly lower than that of fixed-bearing implants [62]. In this cohort, bearing dislocation occurred in 8.5% of patients and the authors emphasize the crucial importance of accurate component alignment in mobile-bearing UKA [62]. Although both fixed- and mobile-bearing implants are widely used for medial UKA, fixed-bearing implants are currently strongly preferred for lateral UKA due to superior survivorship and lower revision rates.

#### Cemented versus cementless implants

Traditionally, lateral UKAs have utilized cement for component fixation with documented long-term success. Cementless components, which have surfaces that encourage bone ingrowth, have emerged as an alternative fixation method. Potential advantages of cementless UKA include shorter procedure length, potentially improved fixation, and avoidance of cementation errors such as loose cement fragments and soft-tissue impingement [63]. However, this may come at the cost of increased risk of intra-operative or early post-operative tibial plateau fracture requiring revision, particularly in elderly patients [64].

Current data on cementless fixation in lateral UKA are limited, though encouraging. Deroche and colleagues found no significant difference in revision-free survivorship between cemented and cementless lateral UKA implants at 10 years, though cemented implants demonstrated superior alignment correction and functional outcomes [54]. Similarly, Kagan and colleagues found similar revision rates (11% versus 8%), patient-reported outcome measures, and patient satisfaction between cemented and cementless lateral UKAs at 10 year follow-up [55]. However, long-term longevity data are still being gathered.

### Survivorship data and outcomes

A growing body of mid- and long-term data offers important insight into survivorship and functional outcomes following lateral UKA, showing favorable outcomes particularly in more contemporary series. There are several cohort studies of lateral UKAs with between five and ten (Table 2) or greater than ten years follow-up (Table 3). These studies have shown mixed but generally high survivorship, with many exceeding 90%, particularly with fixed-bearing designs. A minority of studies used mobile bearings, cementless designs, or robotic-assisted technique. Favroul and colleagues published the longest reported follow-up period of 22.5 years [65]. In this study, survivorship at 25 years was 72.3% across a cohort of lateral UKAs performed between 1988 and 2003. The survivorship and functional outcomes in this series were poorer compared to other, shorter-term studies, which may be due to the older implants used toward the beginning of the series. In 2024, Harkin and colleagues published the largest cohort of lateral UKAs with greater than 10 years mean follow-up to date, including 161 knees with mean follow-up of 10 years, demonstrating 15-year survivorship of 91.3% [66]. Recent registry data show comparable results, with 95.4% survivorship at 7 years with implant revision as the endpoint or 93.5% for combined endpoint of revision or implant addition [67]. Factors affecting implant survivorship include individual variation in tibial slopes, component positioning, and alignment considerations [17].

Lateral UKA has shown similar functional outcomes to medial UKA. A meta-analysis showed no differences in pain score, function score, short to mid-term survivorship (95.6% in medial UKA and 94.6% in lateral UKA) and long-term survivorship (92.8% in medial UKA and 86.6% in lateral UKA) between the two [68]. Recent comparative outcomes show similar Oxford Knee Scores, comparable complication rates, no significant difference in revision rates, and similar functional improvements between medial and lateral UKA [69].

Compared to TKA, lateral UKA affords superior range of motion, faster recovery with shorter hospital stay, improved proprioception, more natural knee kinematics, and higher patient satisfaction with comparable survivorship [66, 70, 71]. Lateral UKA is also associated with lower in-hospital mortality, hospital costs, and risk of certain adverse events including wound infection, blood transfusion, pulmonary embolism, pneumonia, and acute kidney injury [71, 72]. Although the higher long-term revision rates for UKA compared to TKA have raised concerns about durability, recent registry data show that revision rates for lateral UKA have decreased in recent and are approaching that of TKA, especially with the use of newer fixed-bearing implants and improved surgical technique [73].

### Failure and revision of lateral UKA

Overall, the main failure modes of lateral UKA include progression of OA (30%), aseptic loosening (22%), instability (7%), unexplained pain (5%), infection (5%), polyethylene wear (5%), and bearing dislocation (5%), according to a 2018 systematic review [59]. Early failure, defined as failure within 5 years, is most commonly due to bearing dislocation (29%) and OA progression (22%). Mid-term failure (within 5 to 10 years) is most often caused by OA progression (59%), with the second most common cause being fracture (21%). Late-term failure is similarly most often due to OA progression (78%), with less common causes being aseptic loosening (11%) and polyethylene wear (11%) [59]. Mobile- and fixed-bearing implants also have distinct modes of failure. Bearing dislocation (27%) and aseptic loosening (20%) are more common for mobile-bearing implants, while OA progression (44%) is the most common mode of failure for fixed-bearing implants [59]. A separate systematic review also noted that OA progression was the most common mode in cohort studies, whereas aseptic loosening was most common in registry-based studies [74]. This observed difference may be due to slower development of loosening compared to OA progression or loss to follow-up that may have prevented cohort studies from capturing all cases of loosening. Further long-term cohort and registry-based studies are needed to elucidate factors affecting lateral UKA failure.

Failed lateral UKAs are frequently revised to TKA, but revision knee joint systems can also be used. Citak and colleagues reported a mean time to revision surgery of 9.4 years, with the most common indication for revision being OA progression [75]. Revision of UKA to TKA is less technically demanding than revision of primary TKA [76]. Once a UKA is revised to TKA, functional outcomes, complication, and survivorship of such revisions are more comparable to revision TKA than primary TKA. A cohort by Lunebourg and colleagues demonstrated that while primary TKA had 10-year reoperation-free survivorship of 96%, the rate was 72% for revision of UKA and 68% for revision of TKA [76]. These increased revision rates and possibly poorer outcomes of revision from lateral UKA to TKA, compared to primary TKA, may be at least partially due to a lower threshold for revision in UKA than in TKA [77]. A study by Johnson and colleagues found that at revision, patients undergoing revision of UKA had significantly better Oxford Knee Scores and range of motion than those undergoing revision of TKA [78]. However, these and many other published studies analyzing UKA revisions combine medial and lateral UKAs in analysis. Further study is needed to characterize the indications, survivorship, and outcomes of revision lateral UKA specifically.

## Conclusions

Lateral UKA is an effective solution for isolated lateral compartment arthrosis. The distinct anatomy and biomechanics of the lateral compartment require tailored implant design and surgical technique, making it a separate entity from medial UKA. Modern series demonstrate excellent functional outcomes and survivorship, with results comparable to medial UKA and sometimes superior to TKA in select domains such as kinematic restoration and recovery. Fixed-bearing designs and cemented implants are currently preferred, although cementless options have shown promising early outcomes. Robotic-assisted techniques may further refine accuracy and long-term success. Despite these advances, lateral UKA remains underutilized, and further research is needed to clarify optimal patient selection, refine alignment techniques, and characterize failure modes and revision outcomes. Emerging long-term data will determine whether improved component positioning facilitated by robotic assistance can reduce revision rates and enhance patient satisfaction, while ongoing developments in cementless fixation may improve implant survivorship.

**Table 1:** Impact of age, obesity, and patellofemoral joint disease on lateral unicompartmental knee arthroplasty outcomes

Study	Study type	No. of knees	Mean follow-up (yr)	Age (< 60 year)	Obesity	PFJ disease
Baker et al. 2012 [79]	Registry	2052	n.m.	Relative C/I Risk of failure decreases with increasing age	n.m.	n.m.
Burger et al. 2020 [23]	Registry	537	3	Relative C/I < 60 year: 15.4% revision at 5 yr > 60 year: 9.7% revision at 5 yr	Relative C/I BMI < 30: 7.7% revision at 3 yr BMI > 30: 11.5% revision at 3 yr	n.m.
Kennedy et al. 2020 [26]	Cohort	325	7	Not a C/I	Not a C/I	n.m.
Deroche et al. 2020 [54]	Cohort	268	9.1	Relative C/I Non-significant trends for higher risk of revision surgery in younger subjects	Relative C/I Non-significant trends for higher risk of revision surgery in case of high BMI subjects	n.m.
Burger et al. 2020 (1) [80]^a^	Cohort	171	4.3	Not a C/I	Not a C/I	n.m.
Burger et al. 2020 (2) [27]	Cohort	140	4.1	n.m.	n.m.	Not a C/I
Giordano et al. 2023 [25]	Cohort	100	6.5	n.m.	Not a C/I	n.m.
Marullo et al. 2025 [81]	Cohort	96	14.5	n.m.	Not a C/I	n.m.
Heckmann et al. 2022 [82^a^	Cohort	84	4	Not a C/I	Not a C/I	n.m.
Cavaignac et al. 2013 [83]	Cohort	55	12	n.m.	Not a C/I	n.m.
Deroche et al. 2019 [84]	Cohort	54	17.9	Not a C/I	n.m.	n.m.
Fornell et al. 2018 [85]	Cohort	41	4.1	n.m.	Not a C/I	Not a C/I unless deep eburnation and grooving are present
Xing et al. 2012 [86]	Cohort	31	4.5	Not a C/I	Not a C/I	Not a C/I

Survivorship is reported as Kaplan-Meier survivorship if specified in the study. If not specified, it was calculated based on the percentage of successful, revision-free cases.

*PFJ *patellofemoral joint, *C/I* contraindication, *BMI *body mass index, *n.m.* not mentioned

^a^Robotic-assisted surgical technique was used

**Table 2:** Cohort studies on lateral unicompartmental knee arthroplasty with 5 to 10 years of mean Follow-Up

Study	No. of knees	Implant type	Mean age (yr)	Mean follow-up (yr)	Survivorship (time scale)	Indications for revision	Revision surgeries
Kennedy et al. 2020 [26]	325	Mobile, cemented	65	7	85% (10 year)	Dislocation (4.3%) OA progression (3.7%) Infection (0.6%) Recurrent hemarthrosis (0.6%) Aseptic loosening (0.3%) Locking knee (0.3%) Unexplained pain (0.3%)	Bearing exchange (5.2%) TKA (3.7%) Medial UKA (1.5%) Conversion to fixed bearing component (0.3%)
Deroche et al. 2020 [54]	268	Fixed, both cemented (70%) and cementless (30%)	68.8	9.1	85.4% (5 year)	OA progression (9.7%) Tibial loosening (1.5%) Technical error / malpositioning (1.1%) Sepsis (1.1%) Medial condyle osteonecrosis (0.4%) Flexion contracture (0.4%) Continued pain (0.4%)	TKA (10.1%) Medial UKA (1.5%) Lateral vertical patellectomy (0.7%) Femoral component replacement (0.7%) Lavage (0.7%) 2-stage exchange (0.4%) Mobilization under general anesthesia (0.4%)
Xue et al. 2021 [87]	248	Fixed, cemented	70.6	5	99.5% (5 year)	Knee stiffness (1%) OA progression (0.5%)	Manual release (1%) Medial UKA (0.5%)
Fitzsimons et al. 2023 [70]	246	Fixed, cemented	57	6.6	85% (10 year)	OA progression (2%) Medial meniscal tear (2%) Infection (1%) Instability (1%)	TKA (2%) Partial medial meniscectomy (2%)
Liebs and Herzberg 2013 [88]	128	Mobile, cemented	73.6	6	83% (9 year)	Aseptic loosening (4.7%) OA progression (2.3%) Fracture (1.6%) OA progression (0.8%) Arthroscopy (0.8%) Internal fixation (0.8%) Impingement (0.8%)	n.m.
Tu et al. 2020 [71]	121	Fixed, cemented	70.2	5.3	99.2% (5 year)	OA progression (0.8%)	Medial UKA (0.8%)
Pandit et al. 2010 [61]	118 (53 open approach, 65 modified technique)	Mobile, cemented	68 for open 53 for modified	5	Open: 82% (4 year) Modified: 91% (4 year)	Bearing dislocation (11.3% of open; 4.6% of modified) Infection (5.7% of open) OA progression (4.6% of modified) Aseptic loosening (1.9% of open) Tibial stress fracture (1.9% of open) Recurrent hemarthrosis (1.5% of modified) Pigmented villonodular synovitis (1.5% of modified)	Bearing exchange (7.5% of open; 4.6% of modified) TKA (7.5% of open)
Smith et al. 2014 [89]	101	Fixed, cemented	64.8	5.36	95.5% (5 year)	OA progression (1%) Subsidence of tibial component (1%) Infection (1%) Unknown (1%)	TKA (3%)
Giordano et al. 2023 [25]	100	Fixed, cemented	71	6.5	100% (6.5 year)	None	None
Maritan et al. 2023 [47]^a^	95	Fixed, cemented	61	7.5	93% (7.5 year)	Aseptic loosening (2.1%) Periprosthetic fracture (1.1%) Patellofemoral arthritis (1.1%) Continued pain (1.1%)	n.m.
Ashraf et al. 2002 [90]	88	Fixed, cemented	69	9	83% (10 year), 74% (15 year)	OA progression (10.2%) Aseptic loosening (6.8%) Component breakage (4.5%)	TKA (17%)
Yang et al. 2025 [91]	76	Fixed, cemented	68	7	100% (7 year)	None	None
Edmiston et al. 2018 [32]	65	Fixed, cemented	61.3	6.85	94% (7 year)	OA progression (3.1%) Posttraumatic wound dehiscence (1.5%) Continued pain (1.5%)	TKA (6.2%)
Newman et al. 2017 [92]	61	Mobile, cemented	71	7	87% (7 year)	OA progression (4.9%) Continued pain (1.6%) Instability (1.6%) Dislocation (1.6%) Hemarthrosis (1.6%)	TKA (3.3%) Medial UKA (3.3%) Bearing exchange (3.3%) Arthroscopic lavage (1.6%)
Gunther et al. 1996 [93]	53	Mobile, cemented	68	5.2	82% (5 year)	Bearing dislocation (11.3%) Infection, late (5.7%) Aseptic loosening (1.9%) Tibial plateau stress fracture (1.9%)	TKA (11.3%) Bearing exchange (7.5%) Arthrodesis (1.9%)
Hartman et al. 2023 [94]	49	Both fixed and mobile, cemented	54.7	8.8	86.1% (10 year)	n.m.	TKA (8.2%)
Lustig et al. 2011 [95]	49	Fixed, cemented	72.2	8.4	98.1% (10 year)	OA progression (6.1%) Aseptic loosening (2%)	TKA (6.1%) Medial UKA (2%)
Sah and Scott 2007 [12]	48	Mobile, cemented	61	5	100%	None	None
Romagnoli et al. 2020 [35]	26	Fixed, cemented	57.6	9.6	92% (9 year)	Persistent pain (3.8%) Instability (3.8%)	Medial UKA (3.8%) TKA (3.8%)
Ohdera et al. 2001 [38]	18	Fixed, cemented	64.5	5	89% (5 year)	OA progression (5.6%) Aseptic loosening (5.6%)	Lateral UKA revision (5.6%) Medial UKA (5.6%)
Gill and Nicolai 2019 [96]	14	Fixed, cemented	58	5.3	100% (5 year)	None	None

*TKA* total knee arthroplasty,* UKA* unicompartmental knee arthroplasty,* OA* osteoarthritis, *n.m.* not mentioned

^a^Robotic-assisted surgical technique was used

**Table 3:** Cohort studies on lateral unicompartmental knee arthroplasty with at least 10 years of mean Follow-up and indications for revision

Study	No. of knees	Mean age (yr)	Mean follow-up (yr)	Survivorship (time scale)	Indications for revision	Revision surgeries
Harkin et al. 2024 [66]	161	69	10	91.3% (15 year)	OA progression (4.3%) Subsidence of tibial component (0.6%)	TKA (5.0%)
Walker et al. 2025 [62]^a^	115	60.2	13.4	74.8% (10 year)	OA progression (10.4%) Bearing dislocation (8.5%) Persistent pain (4.7%) Aseptic loosening (2.8%) Infection (0.9%) Wound healing disorder (0.9%) Mediolateral instability (0.9%) Broken implant (0.9%)	TKA (22.6%) Mobile bearing exchange (5.7%) Revision to fixed bearing implants (1.9%)
Marullo et al. 2025 [81]	96	64.6	14.5	94.7% (14.5 year)	OA progression (3.1%) Polyethylene wear (1.0%) Aseptic loosening (1.0%)	TKA (4.2%) Medial UKA (1.0%)
Murray et al. 2021 [97]	82	70	14.8	68% (25 year)	OA progression (12.2%) Aseptic loosening (3.7%) Unknown (3.7%) Implant fracture (2.4%)	TKA (20.7%) Revision prosthesis (1.2%) Unknown (1.2%)
Ruderman et al. 2024 [44]^b^	77	62.4	10.2	96.1% (10 year)	OA progression (2.6%) Continued pain (2.6%) Infection (1.3%)	TKA (6.5%) I&D with liner exchange (1.3%)
Plancher et al. 2025 (1) [28]	61	65	10.9	Severe PFJ OA: 18.9 yr No severe PFJ OA: 16.6 yr	n.m.	n.m.
Cartier et al. 1996 [98]	60	65	12	93% (10–12 year)	Infection (5%) Subluxation (5%) OA progression (1.7%)	TKA (5%) Medial UKA (3.3%)
Schmidt et al. 2021 [99]	54	58	10.1	Post-traumatic OA: 64.8% (22 year) Non-traumatic OA: 58.8% (22 year)	OA progression (11.1%) Impingement (3.7%) Aseptic loosening (1.9%) Polyethylene wear (1.9%)	TKA (11.1%) Medial UKA (3.7%) PFJ arthroplasty (1.9%) Revision lateral UKA (1.9%)
Deroche et al. 2019 [84]	54	65.4	17.9	82.1% (15 year), 79.4% (20 year)	OA progression (17.9%) Aseptic loosening (2.6%)	TKA (12.9%) Medial UKA (7.7%)
Lustig et al. 2014 [100]	54	72.2	14.2	86.0% (15 year)	OA progression (11.1%) Aseptic loosening (1.9%)	TKA (7.4%) Medial UKA (5.6%)
Heyse et al. 2012 [101]	50	53.7	10.8	91.8% (10 year), 91.7% (15 year)	n.m.	n.m.
Plancher et al. 2025 (2) [30]	48	67	11	ACL-deficient: 85% (10 year) ACL-intact: 100% (10 year)	Technical error (2.1%) Fall (2.1%)	TKA (4.2%)
Argenson et al. 2008 [102]	40	61	12.6	92% (10 year), 84% (16 year)	OA progression (10%) Tibial loosening (2.5%) Patellar fracture (2.5%)	TKA (7.5%) Medial UKA (2.5%) PFJ arthroplasty (2.5%)
Sangaletti et al. 2024 [24]	40	57.6	11.1	93.1% (10 year)	OA progression (7.5%)	TKA (7.5%)
Deroche et al. 2020 [54]	39	65.4	17.9	82.1% (15 year), 79.4% (20 year)	OA progression (17.9%) Aseptic loosening (2.6%)	TKA (12.8%) Medial UKA (7.7%)
Argenson et al. 2008 [102]	39	61	12.6	92% (10 year), 84% (16 year)	OA progression (10.2%)	Medial UKA (5.1%) PFJ arthroplasty (2.6%) TKA (7.7%)
Kagan et al. 2020 [55]^c^	30	69	10	93% (10 year)	OA progression (3.3%) Unknown (3.3%)	TKA (6.7%)
Pennington et al. 2006 [103]	29	68	12.4	100% (15 year)	None	None
Favroul et al. 2024 [65]	28	68.6	22.5	72.3% (25 year)	OA progression (17.9%) Aseptic loosening (7.1%) Polyethylene wear (3.6%)	TKA (25%) Medial UKA (3.6%)

Survivorship is reported as Kaplan-Meier survivorship if specified in the study. If not specified, it was calculated based on the percentage of successful, revision-free cases. Percentages of total knees meeting each indication for revision and undergoing each type of revision surgery are listed if specified in the study. All studies used fixed bearings and cemented implants unless otherwise specified

*TKA* total knee arthroplasty,* UKA* unicompartmental knee arthroplasty,* I&D* incision and drainage,* PFJ * patellofemoral joint,* OA* osteoarthritis

^a^Mobile bearing was used

^b^Robotic-assisted surgical technique was used

^c^Both cemented and cementless implants were used

## Data Availability

No datasets were generated or analysed during the current study.

## References

[1] National Joint Registry Annual Reports (2019) National Joint Registry

[2] Scott RD (2005) Lateral unicompartmental replacement: a road less traveled. Orthopedics 28(9):983–984. 10.3928/0147-7447-20050901-34 16190078 10.3928/0147-7447-20050901-34

[3] Berend KR, Turnbull NJ, Howell RE, Lombardi AV (2015) The current trends for lateral unicondylar knee arthroplasty. Orthop Clin North Am 46(2):177–184. 10.1016/j.ocl.2014.10.001 25771313 10.1016/j.ocl.2014.10.001

[4] Walker T, Gotterbarm T, Bruckner T, Merle C, Streit MR (2014) Total versus unicompartmental knee replacement for isolated lateral osteoarthritis: a matched-pairs study. Int Orthop 38(11):2259–2264. 10.1007/s00264-014-2473-0 25112651 10.1007/s00264-014-2473-0

[5] van der List JP, Chawla H, Zuiderbaan HA, Pearle AD (2016) Patients with isolated lateral osteoarthritis: unicompartmental or total knee arthroplasty? Knee 23(6):968–974. 10.1016/j.knee.2016.06.007 27810429 10.1016/j.knee.2016.06.007

[6] Robinson BJ, Rees JL, Price AJ, Beard DJ, Murray DM (2002) A kinematic study of lateral unicompartmental arthroplasty. Knee 9(3):237–240. 10.1016/S0968-0160(02)00039-X 12126685 10.1016/s0968-0160(02)00039-x

[7] Sanchez ARI, Sugalski MT, LaPrade RF (2006) Anatomy and biomechanics of the lateral side of the knee. Sports Med Arthrosc Rev 14(1):2 17135939 10.1097/00132585-200603000-00002

[8] Cicuttini FM, Wluka AE, Wang Y, Davis SR, Hankin J, Ebeling P (2002) Compartment differences in knee cartilage volume in healthy adults. J Rheumatol 29(3):554–556 11908572

[9] LaPrade RF, Bollom TS, Wentorf FA, Wills NJ, Meister K (2005) Mechanical properties of the posterolateral structures of the knee. Am J Sports Med 33(9):1386–1391. 10.1177/0363546504274143 16002488 10.1177/0363546504274143

[10] Zingde SM, Slamin J (2017) Biomechanics of the knee joint, as they relate to arthroplasty. Orthop Trauma 31(1):1–7. 10.1016/j.mporth.2016.10.001

[11] Flandry F, Hommel G (2011) Normal anatomy and biomechanics of the knee. Sports Med Arthrosc Rev 19(2):82. 10.1097/JSA.0b013e318210c0aa 21540705 10.1097/JSA.0b013e318210c0aa

[12] Sah AP, Scott RD (2007) Lateral unicompartmental knee arthroplasty through a medial approach: study with an average Five-Year Follow-up. JBJS 89(9):1948. 10.2106/JBJS.F.01457 10.2106/JBJS.F.01457 17768191

[13] Matsuda S, Miura H, Nagamine R et al (2004) Anatomical analysis of the femoral condyle in normal and Osteoarthritic knees. J Orthop Res 22(1):104–109. 10.1016/S0736-0266(03)00134-7 14656667 10.1016/S0736-0266(03)00134-7

[14] Khan FA, Koff MF, Noiseux NO et al (2008) Effect of local alignment on compartmental patterns of knee osteoarthritis. JBJS 90(9):1961. 10.2106/JBJS.G.00633 10.2106/JBJS.G.00633 PMC2663322 18762657

[15] Scott CEH, Nutton RW, Biant LC (2013) Lateral compartment osteoarthritis of the knee: biomechanics and surgical management of end-stage disease. Bone Jt J 95–B(4):436–444. 10.1302/0301-620x.95b4.30536 10.1302/0301-620X.95B4.30536 23539693

[16] Englund M, Lohmander LS (2004) Risk factors for symptomatic knee osteoarthritis fifteen to twenty-two years after meniscectomy. Arthritis Rheum 50(9):2811–2819. 10.1002/art.20489 15457449 10.1002/art.20489

[17] Calek AK, Hochreiter B, Hess S et al (2022) High inter- and intraindividual differences in medial and lateral posterior tibial slope are not reproduced accurately by conventional TKA alignment techniques. Knee Surg Sports Traumatol Arthrosc 30(3):882–889. 10.1007/s00167-021-06477-z 33547913 10.1007/s00167-021-06477-z

[18] Meier M, Zingde S, Best R, Schroeder L, Beckmann J, Steinert AF (2020) High variability of proximal tibial asymmetry and slope: a CT data analysis of 15,807 Osteoarthritic knees before TKA. Knee Surg Sports Traumatol Arthrosc 28(4):1105–1112. 10.1007/s00167-019-05728-4 31570962 10.1007/s00167-019-05728-4

[19] Hernigou P, Deschamps G (2004) Posterior slope of the tibial implant and the outcome of unicompartmental knee arthroplasty. JBJS 86(3):506 10.2106/00004623-200403000-00007 14996875

[20] Skolnick MD, Bryan RS, Peterson LFA (1975) Unicompartmental polycentric knee arthroplasty description and preliminary results. Clin Orthop Relat Res 112:208 1192634

[21] Kozinn SC, Scott R (1989) Unicondylar knee arthroplasty. JBJS 71(1):145 2643607

[22] Pandit H, Jenkins C, Gill HS et al (2011) Unnecessary contraindications for mobile-bearing unicompartmental knee replacement. J Bone Joint Surg Br 93(5):622–628. 10.1302/0301-620X.93B5.26214 21511927 10.1302/0301-620X.93B5.26214

[23] Burger JA, Kleeblad LJ, Sierevelt IN et al (2020) A comprehensive evaluation of lateral unicompartmental knee arthroplasty short to Mid-Term Survivorship, and the effect of patient and implant characteristics: an analysis of data from the Dutch arthroplasty register. J Arthroplasty 35(7):1813–1818. 10.1016/j.arth.2020.02.027 32192831 10.1016/j.arth.2020.02.027

[24] Sangaletti R, Andriollo L, Montagna A, Are L, Benazzo F, Rossi SMP (2024) Lateral UKA can be a safe solution in a young patients’ population: a 10-year follow-up report. Arch Orthop Trauma Surg 10.1007/s00402-023-05189-y 10.1007/s00402-023-05189-y 38231208

[25] Giordano L, Maffulli N, Morenghi E et al (2023) A BMI above 30 results in satisfying outcomes in patients undergoing fixed-bearing lateral unicompartmental knee arthroplasty. Knee Surg Sports Traumatol Arthrosc 31(3):1106–1112. 10.1007/s00167-022-07253-3 36478285 10.1007/s00167-022-07253-3 PMC9734769

[26] Kennedy JA, Mohammad HR, Yang I et al (2020) Oxford domed lateral unicompartmental knee arthroplasty: ten-year survival and seven-year clinical outcome. Bone Jt J 102–B(8):1033–1040. 10.1302/0301-620X.102B8.BJJ-2019-1330.R2 10.1302/0301-620X.102B8.BJJ-2019-1330.R2 32731833

[27] Burger JA, Dooley MS, Kleeblad LJ, Zuiderbaan HA, Pearle AD (2020) What is the impact of patellofemoral joint degeneration and malalignment on patient-reported outcomes after lateral unicompartmental knee arthroplasty? Bone Jt J 102–B(6):727–735. 10.1302/0301-620X.102B6.BJJ-2019-1429.R1 10.1302/0301-620X.102B6.BJJ-2019-1429.R1 32475250

[28] Plancher KD, Comulada DB, DiVella MF et al (2025) Severe lateral facet patella osteoarthritis is not associated with increased failure at mean 10 years after lateral unicompartmental knee arthroplasty. J Arthroplasty 40(2):359–366. 10.1016/j.arth.2024.08.004 39128779 10.1016/j.arth.2024.08.004

[29] Plancher KD, Briggs KK, Brite JE, Petterson SC (2022) The Lawrence D. Dorr surgical techniques & technologies award: patient acceptable symptom state (PASS) in medial and lateral unicompartmental knee arthroplasty: does the status of the ACL impact outcomes? J Arthroplasty 37(8, Supplement):S710–S715. 10.1016/j.arth.2022.01.081 35122945 10.1016/j.arth.2022.01.081

[30] Plancher KD, Briggs KK, Comulada DB et al (2025) Fixed-Bearing lateral unicompartment knee arthroplasty in degenerative ACL-Deficient and ACL-Intact knees: A matched pair analysis. J Arthroplasty 40(1):70–74. 10.1016/j.arth.2024.07.024 39047920 10.1016/j.arth.2024.07.024

[31] Tashiro Y, Matsuda S, Okazaki K, Mizu-uchi H, Kuwashima U, Iwamoto Y (2014) The coronal alignment after medial unicompartmental knee arthroplasty can be predicted: usefulness of full-length valgus stress radiography for evaluating correctability. Knee Surg Sports Traumatol Arthrosc 22(12):3142–3149. 10.1007/s00167-014-3248-2 25155051 10.1007/s00167-014-3248-2

[32] Edmiston TA, Manista GC, Courtney PM, Sporer SM, Della Valle CJ, Levine BR (2018) Clinical outcomes and survivorship of lateral unicompartmental knee arthroplasty: does surgical approach matter? J Arthroplasty 33(2):362–365. 10.1016/j.arth.2017.09.009 29033153 10.1016/j.arth.2017.09.009

[33] Fujita M, Hiranaka T, Mai B et al (2021) External rotation of the tibial component should be avoided in lateral unicompartmental knee arthroplasty. Knee 30:70–77. 10.1016/j.knee.2021.03.016 33873088 10.1016/j.knee.2021.03.016

[34] Scuderi GR, Renner L, Gwinner C, von Roth P, Perka C (2019) Revision of partial knee arthroplasty. In: Argenson JNA, Dalury DF (eds) Partial knee arthroplasty. Springer International Publishing, pp 111–121. 10.1007/978-3-319-94250-6_12

[35] Romagnoli S, Vitale JA, Marullo M (2020) Outcomes of lateral unicompartmental knee arthroplasty in post-traumatic osteoarthritis, a retrospective comparative study. Int Orthop 44(11):2321–2328. 10.1007/s00264-020-04665-z 32561964 10.1007/s00264-020-04665-z

[36] van der List JP, Chawla H, Villa JC, Zuiderbaan HA, Pearle AD (2017) Early functional outcome after lateral UKA is sensitive to postoperative lower limb alignment. Knee Surg Sports Traumatol Arthrosc 25(3):687–693. 10.1007/s00167-015-3877-0 26611898 10.1007/s00167-015-3877-0

[37] Zheng T, Liu D, Chu Z et al (2024) Effect of lower limb alignment on outcome after lateral unicompartmental knee arthroplasty: a retrospective study. BMC Musculoskelet Disord 25(1):82. 10.1186/s12891-024-07208-4 38245762 10.1186/s12891-024-07208-4 PMC10799503

[38] Ohdera T, Tokunaga J, Kobayashi A (2001) Unicompartmental knee arthroplasty for lateral gonarthrosis: midterm results. J Arthroplasty 16(2):196–200. 10.1054/arth.2001.2090 11222893 10.1054/arth.2001.2090

[39] Vossen RJM, ten Noever de Brauw GV, Ruderman LV et al (2024) Large variance in a lateral Osteoarthritic population prior to and following lateral unicompartmental arthroplasty: an analysis of knee phenotypes. Knee 49:97–107. 10.1016/j.knee.2024.05.010 38878673 10.1016/j.knee.2024.05.010

[40] Khamaisy S, Gladnick BP, Nam D, Reinhardt KR, Heyse TJ, Pearle AD (2015) Lower limb alignment control: is it more challenging in lateral compared to medial unicondylar knee arthroplasty? Knee 22(4):347–350. 10.1016/j.knee.2015.02.018 25805084 10.1016/j.knee.2015.02.018

[41] Lonner JH, Klement MR (2019) Robotic-assisted medial unicompartmental knee arthroplasty: options and outcomes. J Am Acad Orthop Surg 27(5):e207–e214. 10.5435/JAAOS-D-17-00710 30289796 10.5435/JAAOS-D-17-00710

[42] Christ AB, Pearle AD, Mayman DJ, Haas SB (2018) Robotic-Assisted unicompartmental knee arthroplasty: State-of-the Art and review of the literature. J Arthroplasty 33(7):1994–2001. 10.1016/j.arth.2018.01.050 29555499 10.1016/j.arth.2018.01.050

[43] Negrín R, Ferrer G, Iñiguez M et al (2021) Robotic-assisted surgery in medial unicompartmental knee arthroplasty: does it improve the precision of the surgery and its clinical outcomes? Systematic review. J Robot Surg 15(2):165–177. 10.1007/s11701-020-01162-8 33111233 10.1007/s11701-020-01162-8

[44] Ruderman LV, Bayoumi T, ten, Noever de Brauw GV, Lan R, Nguyen JT, Pearle AD (2024) Robotic-arm-assisted lateral unicompartmental knee arthroplasty leads to high implant survival and patient satisfaction at mean 10-year follow-up. Knee Surg Sports Traumatol Arthrosc 32(9):2297–2308. 10.1002/ksa.12237 10.1002/ksa.12237 38738827

[45] Zambianchi F, Franceschi G, Rivi E et al (2020) Clinical results and short-term survivorship of robotic-arm-assisted medial and lateral unicompartmental knee arthroplasty. Knee Surg Sports Traumatol Arthrosc 28(5):1551–1559. 10.1007/s00167-019-05566-4 31218389 10.1007/s00167-019-05566-4

[46] Batailler C, White N, Ranaldi FM, Neyret P, Servien E, Lustig S (2019) Improved implant position and lower revision rate with robotic-assisted unicompartmental knee arthroplasty. Knee Surg Sports Traumatol Arthrosc 27(4):1232–1240. 10.1007/s00167-018-5081-5 30066017 10.1007/s00167-018-5081-5

[47] Maritan G, Franceschi G, Nardacchione R et al (2023) Similar survivorship at the 5-year follow-up comparing robotic-assisted and conventional lateral unicompartmental knee arthroplasty. Knee Surg Sports Traumatol Arthrosc 31(3):1063–1071. 10.1007/s00167-022-07218-6 36374325 10.1007/s00167-022-07218-6 PMC9958141

[48] Canetti R, Batailler C, Bankhead C, Neyret P, Servien E, Lustig S (2018) Faster return to sport after robotic-assisted lateral unicompartmental knee arthroplasty: a comparative study. Arch Orthop Trauma Surg 138(12):1765–1771. 10.1007/s00402-018-3042-6 30242566 10.1007/s00402-018-3042-6

[49] Barrett MC, Wilkinson FO, Blom AW, Whitehouse MR, Kunutsor SK (2021) Incidence, Temporal trends and potential risk factors for aseptic loosening following primary unicompartmental knee arthroplasty: A meta-analysis of 96,294 knees. Knee 31:28–38. 10.1016/j.knee.2021.04.005 34111799 10.1016/j.knee.2021.04.005

[50] Batailler C, White N, Ranaldi FM, Neyret P, Servien E, Lustig S (2019) Improved implant position and lower revision rate with robotic-assisted unicompartmental knee arthroplasty. Knee Surg Sports Traumatol Arthrosc Off J ESSKA 27(4):1232–1240. 10.1007/s00167-018-5081-5 10.1007/s00167-018-5081-5 30066017

[51] Zambianchi F, Franceschi G, Banchelli F, Marcovigi A, Ensini A, Catani F (2022) Robotic Arm-Assisted lateral unicompartmental knee arthroplasty: how are components aligned? J Knee Surg 35(11):1214–1222. 10.1055/s-0040-1722346 33511590 10.1055/s-0040-1722346

[52] Favroul C, Batailler C, Canetti R et al (2023) Image-based robotic unicompartmental knee arthroplasty allowed to match the rotation of the tibial implant with the native kinematic knee alignment. Int Orthop 47(2):519–526. 10.1007/s00264-022-05637-1 36422703 10.1007/s00264-022-05637-1

[53] Fratini S, Meena A, Alesi D, Cammisa E, Zaffagnini S, Marcheggiani Muccioli GM (2022) Does implant design influence failure rate of lateral unicompartmental knee arthroplasty? A Meta-Analysis. J Arthroplasty 37(5):985–992e3. 10.1016/j.arth.2022.01.068 35121088 10.1016/j.arth.2022.01.068

[54] Deroche E, Martres S, Ollivier M et al (2020) Excellent outcomes for lateral unicompartmental knee arthroplasty: multicenter 268-case series at 5 to 23 years’ follow-up. Orthop Traumatol Surg Res 106(5):907–913. 10.1016/j.otsr.2020.03.019 32631712 10.1016/j.otsr.2020.03.019

[55] Kagan R, Anderson MB, Bailey T, Hofmann AA, Pelt CE, Ten-Year Survivorship (2020) Patient-Reported Outcomes, and satisfaction of a Fixed-Bearing unicompartmental knee arthroplasty. Arthroplasty Today 6(2):267–273. 10.1016/j.artd.2020.02.016 32577476 10.1016/j.artd.2020.02.016 PMC7303483

[56] Argenson JNA, Parratte S (2006) The unicompartmental knee: design and technical considerations in minimizing wear. Clin Orthop 452:137–142. 10.1097/01.blo.0000229358.19867.60 16906108 10.1097/01.blo.0000229358.19867.60

[57] Kretzer JP, Jakubowitz E, Reinders J et al (2011) Wear analysis of unicondylar mobile bearing and fixed bearing knee systems: a knee simulator study. Acta Biomater 7(2):710–715. 10.1016/j.actbio.2010.09.031 20883831 10.1016/j.actbio.2010.09.031

[58] Kwon OR, Kang KT, Son J et al (2014) Biomechanical comparison of fixed- and mobile-bearing for unicomparmental knee arthroplasty using finite element analysis. J Orthop Res Off Publ Orthop Res Soc 32(2):338–345. 10.1002/jor.22499 10.1002/jor.22499 24122942

[59] Ernstbrunner L, Imam MA, Andronic O, Perz T, Wieser K, Fucentese SF (2018) Lateral unicompartmental knee replacement: a systematic review of reasons for failure. Int Orthop 42(8):1827–1833. 10.1007/s00264-017-3662-4 29030653 10.1007/s00264-017-3662-4

[60] Abu Al-Rub Z, Lamb JN, West RM, Yang X, Hu Y, Pandit HG (2020) Survivorship of fixed vs mobile bearing unicompartmental knee replacement: A systematic review and meta-analysis of sixty-four studies and National joint registries. Knee 27(5):1635–1644. 10.1016/j.knee.2020.09.004 33010783 10.1016/j.knee.2020.09.004

[61] Pandit H, Jenkins C, Beard DJ et al (2010) Mobile bearing dislocation in lateral unicompartmental knee replacement. Knee 17(6):392–397. 10.1016/j.knee.2009.10.007 19919897 10.1016/j.knee.2009.10.007

[62] Walker T, Freericks J, Mick P et al (2025) Long-term results of lateral unicompartmental knee arthroplasty with a mobile-bearing device. Bone Jt J 107–B(3):322–328. 10.1302/0301-620X.107B3.BJJ-2024-0859.R1 10.1302/0301-620X.107B3.BJJ-2024-0859.R1 40020717

[63] Za P, Papalia GF, Cardile U et al (2025) Cementless unicompartmental knee arthroplasty is safe and effective at a minimum follow-up of 4.2 years: A systematic review. J Exp Orthop 12(2):e70253. 10.1002/jeo2.70253 40337672 10.1002/jeo2.70253 PMC12056710

[64] Panzram B, Barbian F, Reiner T, Hariri M, Renkawitz T, Walker T (2023) Clinical and functional results of cementless unicompartmental knee arthroplasty with a minimum follow up of 5 Years-A consecutive cohort of 201 patients. J Clin Med 12(4):1694. 10.3390/jcm12041694 36836231 10.3390/jcm12041694 PMC9966646

[65] Favroul C, Batailler C, Thouvenin C et al (2024) Long-term functional success and robust implant survival in lateral unicompartmental knee arthroplasty: A case series with a mean follow-up of Twenty two and a half years. Int Orthop 48(7):1761–1769. 10.1007/s00264-024-06215-3 38743298 10.1007/s00264-024-06215-3

[66] Harkin W, Kurina S, Berger A et al (2024) Clinical outcomes and survivorship of lateral unicompartmental knee arthroplasty: A large single surgeon cohort. J Arthroplasty 10.1016/j.arth.2024.05.067 10.1016/j.arth.2024.05.067 38823515

[67] Bunyoz KI, Gromov K, Troelsen A (2025) Starting up a lateral unicompartmental knee arthroplasty Practice – Is outcome affected? J Arthroplasty 40(1):22–27e1. 10.1016/j.arth.2024.07.005 39002768 10.1016/j.arth.2024.07.005

[68] Han SB, Lee SS, Kim KH, Im JT, Park PS, Shin YS (2020) Survival of medial versus lateral unicompartmental knee arthroplasty: A meta-analysis. PLoS ONE 15(1):e0228150. 10.1371/journal.pone.0228150 31978110 10.1371/journal.pone.0228150 PMC6980580

[69] Migliorini F, Cocconi F, Prinz J, Ursino N, Mangiavini L, D’Ambrosi R (2023) No difference in Oxford knee score between medial and lateral unicompartmental knee arthroplasty after two years of follow-up: a clinical trial. J Exp Orthop 10(1):134. 10.1186/s40634-023-00704-x 38062183 10.1186/s40634-023-00704-x PMC10703761

[70] Fitzsimons M, van der Stok J, Queally JM, O’Donnell T (2023) Fixed-Bearing unicompartmental knee arthroplasty of the lateral compartment: A series of 246 cases. Arthroplasty Today 23:101183. 10.1016/j.artd.2023.101183 37731595 10.1016/j.artd.2023.101183 PMC10507187

[71] Tu Y, Ma T, Wen T, Yang T, Xue L, Xue H (2020) Does unicompartmental knee replacement offer improved clinical advantages over total knee replacement in the treatment of isolated lateral osteoarthritis? A matched cohort analysis from an independent center. J Arthroplasty 35(8):2016–2021. 10.1016/j.arth.2020.03.021 32265142 10.1016/j.arth.2020.03.021

[72] Maman D, Mahamid A, Yonai Y, Berkovich Y (2024) Comparing complication Rates, Costs, and length of stay between unicompartmental and total knee arthroplasty: insights from a big data analysis using the National inpatient sample dataset. J Clin Med 13(13):3888. 10.3390/jcm13133888 38999453 10.3390/jcm13133888 PMC11242701

[73] Bunyoz KI, Lindberg-Larsen M, Gromov K, Troelsen A (2025) Optimising outcomes in lateral unicompartmental knee arthroplasty: analysing 25 years of registry data. Knee Surg Sports Traumatol Arthrosc Off J ESSKA 10.1002/ksa.12785 10.1002/ksa.12785 PMC12684322 40652369

[74] van der List JP, Zuiderbaan HA, Pearle AD (2016) Why do lateral unicompartmental knee arthroplasties fail today? Am J Orthop Belle Mead NJ 45(7):432–462 28005097

[75] Citak M, Cross MB, Gehrke T, Dersch K, Kendoff D (2015) Modes of failure and revision of failed lateral unicompartmental knee arthroplasties. Knee 22(4):338–340. 10.1016/j.knee.2015.03.008 25887341 10.1016/j.knee.2015.03.008

[76] Lunebourg A, Parratte S, Ollivier M, Abdel MP, Argenson JNA (2015) Are revisions of unicompartmental knee arthroplasties more like a primary or revision TKA? J Arthroplasty 30(11):1985–1989. 10.1016/j.arth.2015.05.042 26100472 10.1016/j.arth.2015.05.042

[77] Tay ML, Monk AP, Frampton CM, Hooper GJ, Young SW (2023) A comparison of clinical thresholds for revision following total and unicompartmental knee arthroplasty. Bone Jt J 105–B(3):269–276. 10.1302/0301-620X.105B3.BJJ-2022-0872.R2 10.1302/0301-620X.105B3.BJJ-2022-0872.R2 36854342

[78] Johnson WB, Engh CA, Parks NL, Hamilton WG, Ho PH, Fricka KB (2020) A lower threshold for revision of aseptic unicompartmental vs total knee arthroplasty. Bone Jt J 102–B(6SuppleA):91–95. 10.1302/0301-620X.102B6.BJJ-2019-1538.R1 10.1302/0301-620X.102B6.BJJ-2019-1538.R1 32475288

[79] Baker PN, Jameson SS, Deehan DJ, Gregg PJ, Porter M, Tucker K (2012) Mid-term equivalent survival of medial and lateral unicondylar knee replacement: an analysis of data from a National joint registry. J Bone Joint Surg Br 94–B(12):1641–1648. 10.1302/0301-620X.94B12.29416 10.1302/0301-620X.94B12.29416 23188905

[80] Burger JA, Kleeblad LJ, Laas N, Pearle AD (2020) Mid-term survivorship and patient-reported outcomes of robotic-arm assisted partial knee arthroplasty: a single-surgeon study of 1,018 knees. Bone Jt J 102–B(1):108–116. 10.1302/0301-620X.102B1.BJJ-2019-0510.R1 10.1302/0301-620X.102B1.BJJ-2019-0510.R1 31888356

[81] Marullo M, Petrillo S, Russo A, Romagnoli S (2025) Long-Term excellent clinical Outcomes, high Survivorship, and low osteoarthritis progression in lateral unicompartmental knee arthroplasty: A 10-Year minimum Follow-Up. J Clin Med 14(7):2492. 10.3390/jcm14072492 40217941 10.3390/jcm14072492 PMC11989692

[82] Heckmann ND, Antonios JK, Chen XT et al (2022) Midterm survivorship of Robotic-Assisted lateral unicompartmental knee arthroplasty. J Arthroplasty 37(5):831–836. 10.1016/j.arth.2022.01.023 35065214 10.1016/j.arth.2022.01.023

[83] Cavaignac E, Lafontan V, Reina N et al (2013) Obesity has no adverse effect on the outcome of unicompartmental knee replacement at a minimum follow-up of seven years. Bone Jt J 95–B(8):1064–1068. 10.1302/0301-620X.95B8.31370 10.1302/0301-620X.95B8.31370 23908421

[84] Deroche E, Batailler C, Lording T, Neyret P, Servien E, Lustig S (2019) High survival rate and very low wear of lateral unicompartmental arthroplasty at long term: A case series of 54 cases at a mean Follow-Up of 17 years. J Arthroplasty 34(6):1097–1104. 10.1016/j.arth.2019.01.053 30777626 10.1016/j.arth.2019.01.053

[85] Fornell S, Prada E, Barrena P, García-Mendoza A, Borrego E, Domecq G (2018) Mid-term outcomes of mobile-bearing lateral unicompartmental knee arthroplasty. Knee 25(6):1206–1213. 10.1016/j.knee.2018.05.016 30523797 10.1016/j.knee.2018.05.016

[86] Xing Z, Katz J, Jiranek W (2012) Unicompartmental knee arthroplasty: factors influencing the outcome. J Knee Surg 25:369–374. 10.1055/s-0031-1299666 23150345 10.1055/s-0031-1299666

[87] Xue H, Ma T, Wen T, Yang T, Xue L, Tu Y (2021) Predictors of satisfactory outcomes with Fixed-Bearing lateral unicompartmental knee arthroplasty: up to 7-year Follow-Up. J Arthroplasty 36(3):910–916. 10.1016/j.arth.2020.10.001 33168343 10.1016/j.arth.2020.10.001

[88] Liebs TR, Herzberg W (2013) Better quality of life after medial versus lateral unicondylar knee arthroplasty. Clin Orthop 471(8):2629–2640. 10.1007/s11999-013-2966-y 23568676 10.1007/s11999-013-2966-y PMC3705060

[89] Smith JRA, Robinson JR, Porteous AJ et al (2014) Fixed bearing lateral unicompartmental knee arthroplasty—Short to midterm survivorship and knee scores for 101 prostheses. Knee 21(4):843–847. 10.1016/j.knee.2014.04.003 24831525 10.1016/j.knee.2014.04.003

[90] Ashraf T, Newman JH, Evans RL, Ackroyd CE (2002) Lateral unicompartmental knee replacement: SURVIVORSHIP AND CLINICAL EXPERIENCE OVER 21 YEARS. J Bone Joint Surg Br 84–B(8):1126–1130. 10.1302/0301-620x.84b8.0841126 10.1302/0301-620x.84b8.13447 12463656

[91] Yang T, Xue H, Ma T, Wen T, Xue L, Tu Y (2025) Lateral unicompartmental knee arthroplasty is an effective procedure for lateral post-meniscectomy knee osteoarthritis: a case–control study at a mean 7-year follow-up. J Orthop Surg 20:284. 10.1186/s13018-025-05615-4 10.1186/s13018-025-05615-4 PMC11907932 40087748

[92] Newman SDS, Altuntas A, Alsop H, Cobb JP (2017) Up to 10 year follow-up of the Oxford domed lateral partial knee replacement from an independent centre. Knee 24(6):1414–1421. 10.1016/j.knee.2017.05.001 28974402 10.1016/j.knee.2017.05.001

[93] Gunther TV, Murray DW, Miller R et al (1996) Lateral unicompartmental arthroplasty with the Oxford meniscal knee. Knee 3(1):33–39. 10.1016/0968-0160(96)00208-6

[94] Hartman J, Dobransky J, Dervin GF (2023) Midterm outcomes in lateral unicompartment knee replacement: the effect of patient age and bearing choice. J Knee Surg 36(8):849–856. 10.1055/s-0042-1743497 35263794 10.1055/s-0042-1743497

[95] Lustig S, Elguindy A, Servien E et al (2011) 5- to 16-Year Follow-Up of 54 consecutive lateral unicondylar knee arthroplasties with a Fixed-All polyethylene bearing. J Arthroplasty 26(8):1318–1325. 10.1016/j.arth.2011.01.015 21414745 10.1016/j.arth.2011.01.015

[96] Gill JR, Nicolai P (2019) Clinical results and 12-year survivorship of the physica ZUK unicompartmental knee replacement. Knee 26(3):750–758. 10.1016/j.knee.2019.02.016 30885547 10.1016/j.knee.2019.02.016

[97] Murray JRD, Smith JRA, Bray R, Robinson JR, White P, Porteous AJ (2021) Fixed bearing, all-polyethylene tibia, lateral unicompartmental arthroplasty – A final outcome study with up to 28 year follow-up of a single implant. Knee 29:101–109. 10.1016/j.knee.2020.12.032 33610116 10.1016/j.knee.2020.12.032

[98] Cartier P, Sanouiller JL, Grelsamer RP (1996) Unicompartmental knee arthroplasty surgery: 10-Year minimum follow-up period. J Arthroplasty 11(7):782–788. 10.1016/S0883-5403(96)80177-X 8934317 10.1016/s0883-5403(96)80177-x

[99] Schmidt A, Barnavon T, Lording T et al (2021) Lateral unicompartmental knee arthroplasty is a safe procedure for post-traumatic osteoarthritis after lateral tibial plateau fracture: a case-control study at 10-year follow-up. Knee Surg Sports Traumatol Arthrosc Off J ESSKA 29(11):3654–3663. 10.1007/s00167-020-06359-w 10.1007/s00167-020-06359-w 33165637

[100] Lustig S, Lording T, Frank F, Debette C, Servien E, Neyret P (2014) Progression of medial osteoarthritis and long term results of lateral unicompartmental arthroplasty: 10 to 18 year follow-up of 54 consecutive implants. Knee 21:S26–S32. 10.1016/S0968-0160(14)50006-3 25382364 10.1016/S0968-0160(14)50006-3

[101] Heyse TJ, Khefacha A, Peersman G, Cartier P (2012) Survivorship of UKA in the middle-aged. Knee 19(5):585–591. 10.1016/j.knee.2011.09.002 21962908 10.1016/j.knee.2011.09.002

[102] Argenson JNA, Parratte S, Bertani A, Flecher X, Aubaniac JM (2008) Long-term results with a lateral unicondylar replacement. Clin Orthop 466(11):2686–2693. 10.1007/s11999-008-0351-z 18574650 10.1007/s11999-008-0351-z PMC2565025

[103] Pennington DW, Swienckowski JJ, Lutes WB, Drake GN (2006) Lateral unicompartmental knee arthroplasty: survivorship and technical considerations at an average Follow-Up of 12.4 years. J Arthroplasty 21(1):13–17. 10.1016/j.arth.2004.11.021 16446180 10.1016/j.arth.2004.11.021

